# Qualitative interviews to understand health care providers’ experiences of prescribing licensed peanut oral immunotherapy

**DOI:** 10.1186/s13104-022-06161-6

**Published:** 2022-08-08

**Authors:** Aikaterini Anagnostou, Claire Lawrence, Stephen A. Tilles, Susan Laubach, Sarah M. Donelson, Mohamed Yassine, Anna Nowak-Wegrzyn

**Affiliations:** 1 Baylor College of Medicine and Texas Children’s Hospital, Houston, TX USA; 2 Acaster Lloyd Consulting Ltd., London, UK; 3 Aimmune Therapeutics, A Nestlé Health Science Company, Brisbane, CA USA; 4 Rady Children’s Hospital, University of California San Diego (UCSD), San Diego, CA USA; 5 Grossman School of Medicine, Hassenfeld Children’s Hospital, New York, USA; 6 Department of Pediatrics, Gastroenterology and Nutrition, Collegium Medicum, University of Warmia and Mazury, Olsztyn, Poland

**Keywords:** Oral immunotherapy, Food allergy treatment, Peanut allergy, Peanut oral immunotherapy, Desensitization, Palforzia

## Abstract

**Objective:**

This research sought to explore health care providers’ (HCPs) experiences of delivering the first US Food and Drug Administration (FDA) and European Commission (EC) approved peanut oral immunotherapy (peanut OIT; Palforzia). Semi-structured qualitative interviews with HCPs who had initiated treatment with ≥ 3 patients in the first nine months following FDA approval sought to identify challenges faced and successful implementation strategies.

**Results:**

Eight allergists and three nurse practitioners from eight sites based in the United States participated. The HCPs included in this research were motivated to implement this novel treatment, however, entered the process with some reservations. HCPs described how successful implementation of peanut OIT requires them to be thoughtful about their clinic’s abilities to integrate complex, time-consuming treatments into their daily practice. Prior experience of OIT was deemed beneficial, but not essential for implementation and learning from others’ experience was suggested as a way of helping new prescribers overcome perceived and actual implementation challenges. Delivering licensed peanut OIT during the COVID-19 pandemic posed both challenges and unexpected opportunities for implementation. The experiences described have the potential to benefit the wider allergy community by providing practical solutions, successful implementation strategies and opportunities to enhance training and resources.

**Supplementary Information:**

The online version contains supplementary material available at 10.1186/s13104-022-06161-6.

## Introduction

Peanut allergy (PA) is a common and typically life-long food allergy [1, 2]. PA is associated with considerable patient and family burden [3], including the likelihood of allergic reactions [4], healthcare utilization [4], and quality of life impacts [3, 5–7]. Palforzia^®^ [Peanut (*Arachis hypogaea*) Allergen Powder-dnfp; defatted powder of *Arachis hypogaea* L., semen (peanuts); previously known as AR101; Aimmune Therapeutics, Brisbane, California, USA] is a licensed peanut oral immunotherapy (OIT) [8]. It is indicated for the mitigation of allergic reactions that may occur with accidental exposure in patients (4–17 years) with a confirmed PA [9].

**Fig. 1:**
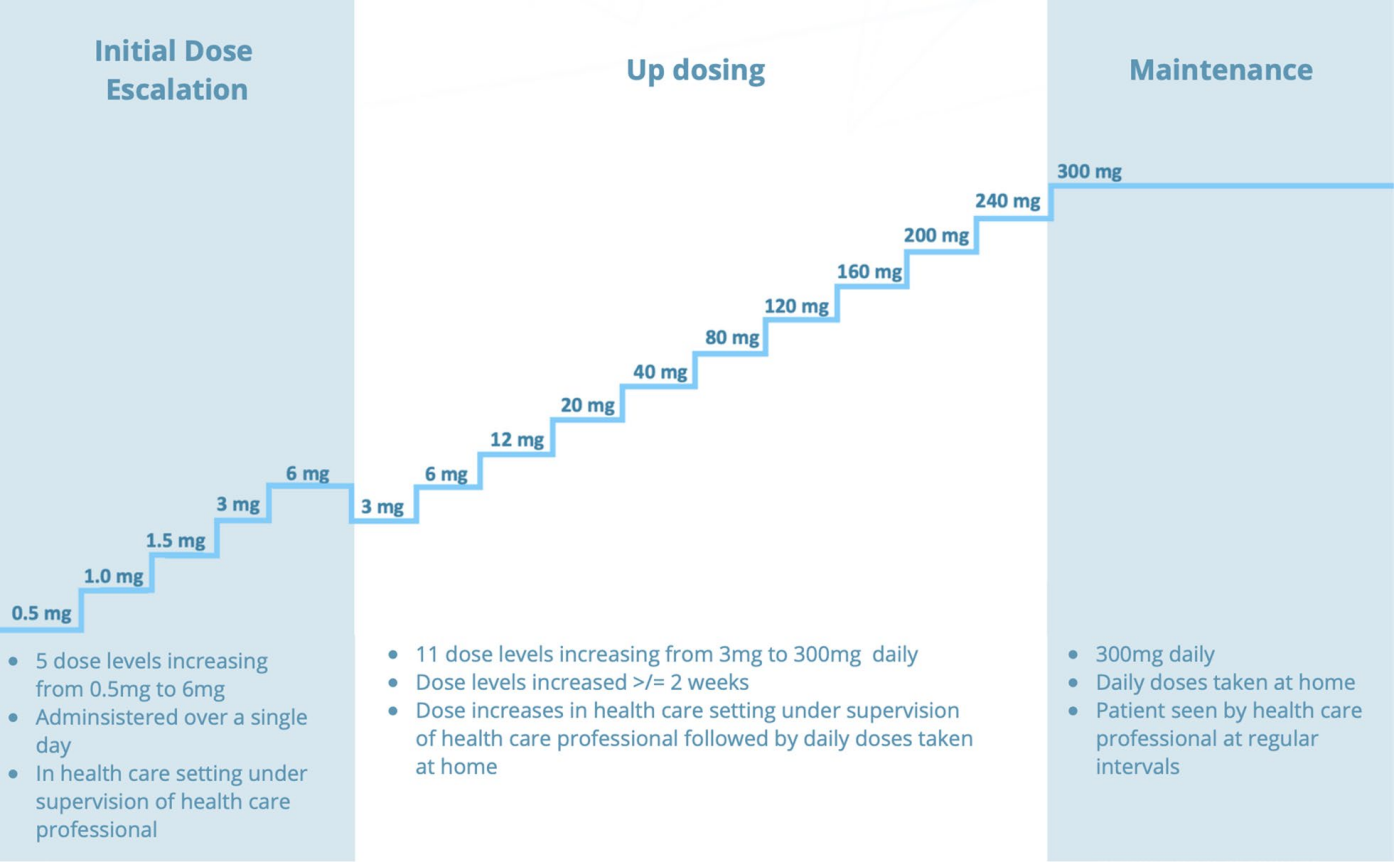
Phases of Palforzia delivery

Treatment involves daily ingestion of precise amounts of peanut protein, gradually increasing over time until reaching a consistent “maintenance” dose (Fig. 1). OIT represents a new treatment paradigm for many allergists [8, 10, 11]. The adoption of any new therapy may require new skills and introduce logistical and practical considerations and potential prescribers may benefit from the experience of others [12, 13]. The primary objective of this study was to better understand the experiences of health care providers (HCPs) in prescribing the only FDA approved treatment for food allergy. An in-depth, qualitative exploration of real-world experiences was conducted. Interviews explored clinical, logistical and administrative considerations, and lessons learned. The experiences described may help inform clinical practice as Palforzia becomes more widely available in the US, Europe and beyond.

## Main text

### Methods

HCPs (allergists, nurse practitioners and physician assistants) prescribing or delivering licensed peanut OIT were eligible to participate if they had been involved in the treatment of at least three patients, practiced in the United States (US), and were willing and able to give written informed consent.

The study sponsor provided a list of all HCPs who had prescribed Palforzia using data from the specialty pharmacy prescription database. Potentially eligible participants were identified and invited to participate by researchers. Eleven HCPs were eligible and willing to participate. The study sponsor was blinded to participants’ identities, although participants were aware of the identity of the study sponsor. The study protocol was reviewed and exempted by the Western Institutional Review Board (Submission Number 2644765-44692237).

Upon written informed consent, participants completed a bespoke background questionnaire assessing their professional background and experience treating food allergy (Additional file 1). Consent was re-confirmed verbally at the start of the 60-min interviews. Interviews were conducted and recorded using Zoom videoconferencing software, transcribed, and de-identified. Transcripts underwent content analysis facilitated by MAXQDA (standard version) [14, 15].

### Sample characteristics

Eight allergists and three nurse practitioners based in the US participated in this study (total n = 11; Table 1). The HCPs treated patients across eight urban allergy practices in seven states located in the Northeast, South, and Mid-West. Six practices were private (8 HCPs) and two were academic (3 HCPs). Participants had a mean of 17 years of experience in food allergy (range 2–40). Five allergists trained in pediatric medicine and three in internal medicine. Two thirds of the sample (64%; n = 7) had prior OIT experience, including HCPs from 3 sites with experience during clinical trials (45%; n = 5).

**Table 1:** Sample characteristics

	% (N)
Professional background	
Allergist/immunologist	73 (8)
Nurse practitioner	27 (3)
Route to allergy (if physician)	
Pediatrics	63 (5)
Internal medicine	38 (3)
Practice type (N = 8)	
Private institution	75 (6)
Academic institution	25 (2)
Prior experience with OIT	64 (7)
Experience with AR101 in trial setting	45 (5)
Experience with off-label peanut OIT	36 (4)
Experience with other food OIT (e.g., milk, egg, tree nuts)	27 (3)

	Mean (range)
Years of experience in food allergy	17 (2–40)
Mean number of licensed peanut OIT patients as of OCT 23, 2020	6 (3–12)

### Qualitative analysis results

**Fig. 2:**
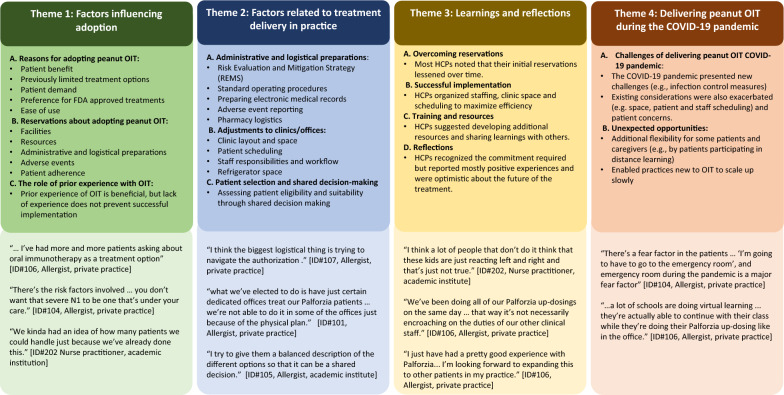
HCP experiences of delivering Palforzia

Experiences were characterized by four key themes: 1. factors influencing adoption, 2. factors related to treatment delivery in practice, 3. learnings and reflections, and 4. delivering Palforzia during the COVID-19 pandemic. An overview of the themes, alongside illustrative quotes, is presented in Fig. 2 and in Additional files 2, 3, 4, 5. Quotes are presented without hesitations (e.g., ‘um’ or ‘uh’) for ease of reading.

## Theme 1: factors influencing adoption

HCPs described reasons for, and reservations about adopting licensed peanut OIT as well as the role of prior experience with OIT. Selected quotes illustrating this theme are presented in Fig. 2 and within Additional file 2.

### Reasons for adopting Palforzia

HCPs described the benefit of treatment to patients both in terms of reducing the risk of serious reactions and potential benefits to patients’ quality of life. HCPs were eager to offer this option to a population where treatments have previously been limited and noted increasing patient demand for peanut OIT. Some HCPs with no previous experience of OIT reported a greater level of comfort using an FDA approved product.

For HCPs with prior experience with OIT, the decision to use licensed peanut OIT was partly driven by hospital policy to use FDA approved treatments, but Palforzia was also perceived as easy to administer with standard, pre-packaged doses.

### Reservations about adopting Palforzia

A minority of participants, all of whom had prior OIT experience, had no reservations about implementation. However, many HCPs without prior OIT experience were concerned about the facilities and resources required to integrate OIT into busy practices. In addition, these HCPs were concerned about administrative and logistical preparations, having enough space, refrigeration, and managing patient and staff scheduling.

HCPs noted reservations about the likelihood of patients experiencing adverse reactions and requirements for patients to follow the dosing schedule and adhere to lifestyle-related restrictions (e.g., physical activity limitations before/after dosing).

### The role of prior experience with OIT

Providers suggested that experience with OIT is beneficial, but lack of experience does not prevent successful implementation. Prior experience was described as comforting for providers and families and helped facilitate conversations between patients and HCPs about treatment. Additionally, experience helped HCPs understand the logistical preparations for delivering licensed peanut OIT and anticipate the frequency and type of adverse reactions that may be experienced. However, HCPs without prior experience of OIT described straightforward implementation processes and dosing schedules, positive experiences of training and support, and the benefits of learning from others with OIT experience.

## Theme 2: factors related to treatment delivery in practice

The HCPs described key considerations for implementing licensed peanut OIT, including administrative and logistical preparations, adjustments to practices, and patient factors. There was substantial heterogeneity in the way these considerations were experienced. Selected quotes illustrating this theme are presented in Fig. 2 and within Additional file 3.

### Administrative and logistical preparations

HCPs described spending significant time negotiating the authorization process. Prior to implementation, HCPs described tasks such as enrolling in the Risk Evaluation and Mitigation Strategy (REMS) program, writing standard operating procedures, preparing electronic medical records, preparing for adverse event reporting procedures and considering pharmacy logistics. A key difference emerged between private and academic institutions, with academic institutions appearing to be less flexible regarding product samples. Samples may be required under certain circumstances, for example if the specialty pharmacy isn’t able to deliver the patient’s next dose on time.

### Adjustments to practices

HCPs needed to rethink clinic space and layout to accommodate the length and number of clinic visits. Practices with prior experience of OIT or oral food challenges were typically well prepared for the space requirements. Clinic space, patient scheduling and staff scheduling were closely related, with space and staffing capacities limiting the number of patients that practices were able to treat at any one time. Large and small practices differed in staffing, size of clinic (e.g., number of patient rooms), and refrigerator space.

### Patient selection and shared decision-making

HCPs described balancing eligibility with patient suitability using shared decision-making. Primarily, HCPs assessed clinical eligibility based on the product indication. HCPs emphasized the importance of being certain of the PA diagnosis, with some conducting oral food challenges if needed for a definitive diagnosis.

Assessing patient suitability was more nuanced. Participants conveyed the importance of having open and honest conversations, informing patients and families of the risks and benefits of treatment, as well as setting clear expectations. Discussions included reiterating the importance of adherence, discussing whether the patients and families would be able to assimilate the treatment into their routines, what treatment reactions to expect, and the possible outcomes of treatment. Anxiety was discussed as both a barrier and facilitator to treatment for patients and families. For some, the anxiety of accidental exposure and adverse reactions is a strong motivator for beginning treatment while others may be too anxious about peanut exposure to consider OIT.

## Theme 3: learnings and reflections

Successful implementation required HCPs to consider their clinic’s abilities to integrate complex, time-consuming treatments into their practice while addressing patients’ needs. The HCPs were able to draw upon their own experiences to provide suggestions about additional training and resources which may help future prescribers. Selected quotes illustrating this theme are presented in Fig. 2 and within the Additional file 4.

### Overcoming reservations

While HCPs reported initial reservations prior to implementation, most noted that reservations lessened over time. For example, the REMS certification process was less onerous than expected and adverse reactions were less common than anticipated. Some reservations did persist, particularly for prescribers without prior experience or prescribers located in smaller clinics. For example, a single allergist clinic was concerned about the ability to manage therapy during busier seasons for their practice. Despite some persistent reservations, all HCPs included in this research were able to successfully implement Palforzia.

### Successful implementation strategies

Strategies were employed to maximize the efficiency of staffing, clinic space and scheduling. Some HCPs preferred not to deliver OIT at the same time as other high-resource activities such as food challenges or allergy shots due to space and staffing limitations.

HCPs described the roles that different members of staff have in the treatment pathway with efficiency gained by allocating certain processes to specific staff members, such as patient communication, patient enrollment, insurance approval, administering doses of the treatment, observing patients during dosing visits, and fielding after hours calls.

Some HCPs dedicated specific rooms and spaces for treatment dosing and subsequent observation, while others allocated specific days to conduct initial dose escalations to overcome space and staffing challenges. A key element of successful implementation involved good patient communication.

### Training and resources

HCPs highlighted several areas where additional resources for patients and caregivers may be beneficial, including paper versions of web materials, dosing schedule handouts, frequently asked questions (FAQs), and the availability of ‘dummy’ dose kits.

Additional resources for staff and practices were suggested, including templates for key documents, standard operating procedures, dosing and patient observation tracking logs, informed consent forms, FAQs, updated information on the prevalence of adverse reactions and how to manage them, guidance on insurance and reimbursement, and guidance on the best way to open capsules. One HCP suggested peer-led training, whereby HCPs from experienced or established practices train providers new to licensed peanut OIT.

### Reflections

Overall, the HCPs were positive about their experiences, however there was recognition of the commitment required to deliver licensed peanut OIT in practice. Many were optimistic about the future of this treatment and it was noted patients and families appear to be satisfied with the treatment, and experiencing its benefits.

## Theme 4: delivering Palforzia during the COVID-19 pandemic

The COVID-19 pandemic presented challenges for HCPs to negotiate, and also offered unexpected opportunities. Selected quotes illustrating this theme are presented in Fig. 2 and within Additional file 5.

### Challenges presented by COVID-19

The timing of the FDA approval meant HCPs were required to implement this novel treatment under challenging circumstances. Infection control measures were necessary (e.g., masks, temperature screening, sanitization, enhanced cleaning procedures, physical distancing), which resulted in reduced patient capacity for some clinics.

The COVID-19 pandemic also exacerbated some patient concerns, such as the management of adverse reactions. HCPs described how patients were more fearful of visiting the emergency room due to the pandemic.

### Unexpected opportunities

The pandemic offered unexpected opportunities for some prescribers such as additional flexibility to some patients and caregivers. Virtual learning allowed patients to fit clinic visits around their school schedule more easily. The COVID-19 pandemic has also allowed practices more time to prepare and the ability to scale up slowly during quieter periods.

## Limitations

This research represents an initial exploration of early Palforzia prescribers’ experiences, therefore there are some notable limitations. Firstly, the sample may represent a subset of highly motivated HCPs. Around two thirds of the sample had prior experience with OIT, and almost half had prior experience in clinical trials. In addition, due to the timing of the research, the interviews were unable to fully explore experiences of treatment maintenance. Finally, although the study sponsor was blind to the identity of participants, participants were aware of the identity of the study sponsor and received monetary reimbursement for their time. Thus, their responses may be influenced by social desirability bias.

## Supplementary Information

**Additional file 1: Questionnaire S1.** Background questionnaire. Description of data: Questionnaire assessing participants’ professional background and experience delivering oral immunotherapy including Palforzia.

**Additional file 2: Table S1.** Factors influencing adoption (Theme 1). Table presenting additional quotes to support the data presented in the manuscript (Theme 1).

**Additional file 3: Table S2.** Factors related to treatment delivery in practice (Theme 2). Table presenting additional quotes to support the data presented in the manuscript (Theme 2).

**Additional file 4: Table S3.** Learnings and reflections (Theme 3). Table presenting additional quotes to support the data presented in the manuscript (Theme 3).

**Additional file 5: Table S4.** Delivering Palforzia during the COVID-19 pandemic (Theme 4). Table presenting additional quotes to support the data presented in the manuscript (Theme 4).

## Data Availability

The datasets generated and analysed during the current study are not publicly available to maintain the anonymity of research participants. Additional data may be available from the corresponding author on reasonable request.

## Abbreviations

EC: European Commission
FAQ: Frequently asked question
FDA: Food and Drug Administration
HCP: Health care provider
OIT: Oral immunotherapy
PA: Peanut allergy
REMS: Risk Evaluation and Mitigation Strategy
US: United States
