# Supplementary material for: Qualitative interviews to understand health care providers’ experiences of prescribing licensed peanut oral immunotherapy
Source: BMC Res Notes. 2022 Aug 8;15:273. doi: 10.1186/s13104-022-06161-6 (PMC9358114; doi:10.1186/s13104-022-06161-6)
Supplement: Supplementary file 1 — Additional file 1: Questionnaire S1. Background questionnaire. Description of data: Questionnaire assessing participants’ professional background and experience delivering oral immunotherapy including Palforzia. [file 13104_2022_6161_MOESM1_ESM.pdf]

Background questionnaire

The following questions are about your professional background and your experience delivering oral immunotherapy including PALFORZIA.

1. What is your profession?
   - ☐ Allergist
   - ☐ Nurse practitioner
   - ☐ Physician's assistant

2. If you are an allergist, what route did you take to become an allergist?
   - ☐ Internal medicine
   - ☐ Pediatric medicine
   - ☐ Other, please specify:

3. How many years of experience do you have in Food Allergy?

4. Prior to the FDA approval of PALFORZIA, were you involved in any trials of this treatment?
   - ☐ Yes
   - ☐ No

5. Prior to the FDA approval of PALFORZIA, did you participate in off-label treatment for peanut allergy using peanut protein/peanut flour?
   - ☐ Yes
   - ☐ No

6. Since the FDA approval of PALFORZIA, approximately how many patients have you enrolled on this treatment?

7. Have you had experience delivering any other forms of oral immunotherapy for the treatment of food allergies?
   - ☐ Yes, please specify:
   - ☐ No
