# Supplementary material for: Qualitative interviews to understand health care providers’ experiences of prescribing licensed peanut oral immunotherapy
Source: BMC Res Notes. 2022 Aug 8;15:273. doi: 10.1186/s13104-022-06161-6 (PMC9358114; doi:10.1186/s13104-022-06161-6)
Supplement: Supplementary file 2 — Additional file 2: Table S1. Factors influencing adoption (Theme 1). Table presenting additional quotes to support the data presented in the manuscript (Theme 1). [file 13104_2022_6161_MOESM2_ESM.pdf]

Additional file 2

Supplemental Table 1. Factors influencing adoption (Theme 1)

Sub-theme	Selected quotes
**Reasons for adopting Palforzia**	*Comfort in using an FDA approved product*<br>"My comfort level has been significantly increased in terms of recommendations as well as treatment of peanut allergy, because of the switch from a kind of do-it-yourself bio-hack if you will to … being out, now the vetting and, and you know, and industrial back-up and you know, being one guy with a nurse practitioner, it's good to have that." [ID#102, Allergist, private practice]<br><br>*Policy to implement FDA approved products*<br>"Once Palforzia became approved, our hospital's policy was that if there's an FDA approved product, we were no longer allowed to use a non-FDA approved product so we, as soon as Palforzia was approved, we had to stop offering peanut flour to our patients and we are only allowed to offer Palforzia, if they want peanut desensitisation." [ID#105, Allergist, academic institute]<br><br>*Ease of delivering Palforziacompared to off-label OIT*<br>"The process of it and the time, the, the way that the doses are delivered, and already divided I think takes out a lot of the pre-work that is required for food oral immunotherapy." [ID#107, Allergist, private practice]<br><br>*Evidence supporting Palforzia*<br>"Ultimately, I like the fact that there's a large amount of data behind the product, so large-scale studies that have been, you know, well designed and have been thoroughly investigated so at, you know, the fact that there is some evidence-based medicine behind this product a large plus and one of the things that makes it attractive for me." [ID#107, Allergist, private practice]<br><br>*Patient benefit*<br><br>"Primarily, to have an option to provide to patients and their families with peanut allergy, uh so that we could help to minimise the risk of a potentially serious allergic reaction from uh, accidental ingestion and thus improving quality of life for many of these families and their, and their children." [ID#107, Allergist, private practice]<br><br>*Previously limited treatment options*<br><br>"No. 1, so that, that's the biggest thing and now that it's been 20 years of not having any option of what to do, is probably the biggest reason." [ID#104, Allergist, private practice]
**Reservations about adopting Palforzia®**	*Adopting a new therapy*<br><br>"Yeah, absolutely, I mean it's a brand-new therapy and so again it's been in clinical trials for several years before it was FDA approved but I didn't have any personal experience with it, so whenever you start a new therapy you're always concerned about the risks and benefits and the actual like day-to-day questions that may come up, … that was concerning.*"* [ID#106, Allergist, private practice]

	Administrative preparations and burden<br><br>"the idea of it [REMS] was more daunting than what it really was." [ID#104, Allergist, private practice]<br><br>*Adverse reactions*<br><br>"There's the risk factors involved with, we don't want to harm a patient and … the percentages are that if you do enough of this, somebody's going to have an adverse reaction, but you don't want that severe N1 to be one that's under your care." [ID#104, Allergist, private practice]<br><br>*Compliance*<br><br>"A concern as well is a lot of the restrictions that are, required on physical activity in and around the time of the dosing and so like, like in my patient population, there are many of the children are involved in sports and things like that and so that is a concern, like are patients going to be compliant with avoiding those activities and things that could potentially reduce the potential for having an anaphylactic reaction while they're on therapy." [ID#106, Allergist, private practice]
**The role of prior experience**	*Comfort for providers and families*<br><br>"I think having had experience is very comforting to families, they like to know that the provider they're seeing has done this before and has had experience." [ID#105, Allergist, academic institute]<br><br>*Discussing Palforzia™ with patients*<br>*"*It made us more comfortable doing it because we had been, we'd had some experience giving peanut flour as well as Palforzia and under our belt, we felt more comfortable offering it to our patients, it was easier to discuss you know, the pros and cons and the risks and benefits to them because we'd experienced it before." [ID#105, Allergist, academic institute]<br><br>*Understanding adverse events*<br><br>"I mean I'm clearly prepared. [laughs] But I also don't anticipate the same frequency of severe reactions or need for Epi." [ID#101, Allergist, private practice]<br><br>*Straightforward implementation*<br><br>"Getting it started wasn't terribly difficult… like the overall process was pretty easy, like the rep for Palforzia was helpful in getting us or walking us through the steps for as far as giving the, the REMS certification for oursel- for, for myself, the practice and then also getting us the office] dose kit and everything like that." [ID#106, Allergist, private practice]<br><br>*Training and support*<br><br>"The medical science liaison also helped us, had a meeting with Dr [Name] who's uh, one of the … the big investigators in the clinical trial and he was also able to answer a lot of our questions that we had with regards to the adoption of Palforzia in some of the, the day-to-day issues or questions or concerns that may come up

	and that also really helped with the adoption of the Palforzia as well." [ID#106, Allergist, private practice]<br><br>*Learning from others*<br><br>"We have a staff member, a physician on our staff who had been doing this off-label with a previous practice and she's really been great at educating everyone on oral immunotherapy and the procedures and getting everybody up to speed." [ID#103, Allergist, private practice]
