# Supplementary material for: Qualitative interviews to understand health care providers’ experiences of prescribing licensed peanut oral immunotherapy
Source: BMC Res Notes. 2022 Aug 8;15:273. doi: 10.1186/s13104-022-06161-6 (PMC9358114; doi:10.1186/s13104-022-06161-6)
Supplement: Supplementary file 3 — Additional file 3: Table S2. Factors related to treatment delivery in practice (Theme 2). Table presenting additional quotes to support the data presented in the manuscript (Theme 2). [file 13104_2022_6161_MOESM3_ESM.pdf]

Additional file 3

Supplemental Table 2. Factors related to treatment delivery in practice (Theme 2)

Sub-theme	Selected quotes
**Administrative and logistical preparations**	*The REMS programme*<br><br>"I mean when all the providers were getting signed up initially, it was like super-easy, yeah we didn't really have any issues with that, … we really had to make sure all of the providers signed up, whether they had any intent of prescribing it in their office or not, just so they would be able to advise a patient after hours or if it was their patient that they were sending to another location to get Palforzia … I mean it was just a matter of getting the doctors and getting everyone to login and do it, it was very easy then." [ID#201, Nurse practitioner, private practice]<br><br>*Preparing SOPs*<br>"I think we started the process like in the late March and then getting everything set up as well… as far as meeting the REMS certification and having like a standard operating procedure for the office as well that was acceptable for Palforzia took about six to eight weeks, I would say." [ID#106, Allergist, private practice]<br><br>"So that's where most of our training pre Palforzia was, I would say for OIT, when we started doing that, unfortunately it was kind of trial by fire, we got protocols from other centres and then we kind of just worked our way through it until we got it to a system that we liked." [ID#202, Nurse practitioner, academic institute]<br><br>*Pharmacy logistics*<br><br>"We have to get approval from the leadership of the hospital as well as our pharmacy had to sign off on it, and somebody else had to sign off on it and now I can't remember. But it, it had to go through a very lengthy process to get it into the hospital and we had to kind of change the language to make it not be a "sample". They still don't like it, but because it's such a small percentage of patients, they're doing it, but it is definitely not what pharmacies, uh hospital pharmacies want to be doing." [ID#202, Nurse practitioner, academic institute]<br><br>"The one issue that still kinda comes up is like with the, some of the specialty pharmacies, we do have to contact them like every other week to make sure that they're sending the Palforzia, unfortunately they're not doing that automatically but that's not a, a major hurdle." [ID#106, Allergist, private practice]
**Adjustments to clinics/offices**	*Patient scheduling*<br><br>"We did not have to make any adjustments although we did have to you know we've had to designate certain days in the clinic that we can particularly provide the initial dose escalation appointment because of some space limitations and staffing limitations. So, we've kind of had to, you know … dictate when this may

	happen based on our clinic space and staffing.” [ID#107, Allergist, private practice]<br><br>*Fridge space*<br><br>“I think challenges, obviously getting the product, I mean keeping it refrigerated, you know, you have to have the space in the fridge, in the refrigerators, we just happen to have a lot of them, we’re, you know we’re a pretty big office so we have a lot of, we’re able to manage that but I can see a small practice not being able to do it, I mean not having enough refrigerator space.” [ID#101, Allergist, private practice]
**Patient factors**	*Patient selection*<br><br>“Generally, it’s any child with peanut allergy is a candidate, the best candidates are kids who have had … a fairly frequent reaction, accidental reactions to peanut and accidental exposures leading to symptoms, they are probably the best candidates, the children have a peanut allergy but haven’t had a reaction in six years and everybody’s doing well with avoidance, they’re not really candi- they’re not going to be interested anyway because you have this whole treatment to prevent reactions that really aren’t happening.” [ID#103, Allergist, private practice]<br><br>*Oral food challenges*<br><br>“In some cases, the child has outgrown their peanut allergy and if their IgE's are really low, we t- just bring them in and do an oral challenge and if they pass, they don’t need to take Palforzia so it’s good, it’s good to know whether you need it or not.” [ID#105, Allergist, academic institute]<br><br>*Shared decision making*<br><br>"… answering all their questions like not only of the parents but also of the child as well because I always try to make sure that it’s something that the child wants to do as well and it’s not something that’s being kind of pushed upon them, because this is a long-term commitment and I want to make sure everyone that’s involved is all on the same page.” [ID#106, Allergist, private practice]<br><br>*Setting expectations*<br>“I definitely make that clear, that this is not intended to be a cure for their peanut allergy and that you know they will start to have their auto injectable epinephrine available at all times and then you know, also talking about the, the commitment that will be involved in terms of you know, the initial dose escalation visit, up-dosing visits.” [ID#107, Allergist, private practice]<br><br>*Discussions with patients*<br>“Many of the children are involved in sports and things like that and so that is a concern, like are patients going to be compliant with avoiding those activities and things that could potentially reduce the potential for having an anaphylactic reaction while they’re on therapy. So those are all things that like went into the consideration of and so I had the, I do extensive counselling with my patients before starting therapy with regards to all of that.” [ID#106, Allergist, private practice]

	Anxiety<br><br>"So the type of patient would probably be that patient that's very anxious, so we'd have some who are like crazy anxious and that would be the type of patient who would like, they would go, they would jump through hoops to been able to have a reassurance that they're not gonna have, you know, they're not gonna die just having an accidental ingestion." [ID#101, Allergist, private practice]
