# Supplementary material for: Qualitative interviews to understand health care providers’ experiences of prescribing licensed peanut oral immunotherapy
Source: BMC Res Notes. 2022 Aug 8;15:273. doi: 10.1186/s13104-022-06161-6 (PMC9358114; doi:10.1186/s13104-022-06161-6)
Supplement: Supplementary file 4 — Additional file 4: Table S3. Learnings and reflections (Theme 3). Table presenting additional quotes to support the data presented in the manuscript (Theme 3). [file 13104_2022_6161_MOESM4_ESM.pdf]

Additional file 4

Supplemental Table 3. Learnings and reflections (Theme 3)

Sub-theme	Selected quotes
**Overcoming reservations**	*REMS*<br><br>"The idea of it was more daunting than what it really was." [ID#104, Allergist, private practice]<br><br>*Resources and logistics (after initial organisation, many processes became straight forward)*<br><br>"It's way easier than I thought it was going to be." [ID#203, Nurse practitioner, private practice]<br><br>*Treatment reactions*<br><br>"I haven't seen the same kinda reactions in the patients although we hadn't really gotten that many up to maintenance dosage but my anticipation is you're gonna see patients who are allergic but in the trial, all the patients had to have reactions at 100 milligrams or less, okay, so those are the most, most allergic patients… I don't anticipate seeing that as high an incidence of reactions because of patient selection." [ID#101, Allergist, private practice]<br><br>*Some reservations persist*<br><br>"As far as the how we do it, our office, especially in the earlier part when we started, we were quieter because of the pandemic so I don't know when we get more normal again, of especially spring time in our area is very busy in the office so I don't know if I'm going to have to make changes and my questions going forward are going to be how many can a small guy like me handle at one point, and not disrupt my office?" [ID#104, Allergist, private practice]
**Successful implementation**	*Delivering Palforziaalongside other clinic activities*<br><br>"In a clinic where we are doing several food challenges a week, I think we're very comfortable with managing anaphylaxis in the office and managing allergic reactions, you know maybe if it's not anaphylaxis but even more minor allergic reactions in the clinic and I think our staff is able and trained to do that." [ID#107, Allergist, private practice]<br><br>"From a space and staffing standpoint, to do a new start Palforzia initial dose escalation and a food challenge on the same day, so we're trying to you know, separate those two very time intensive and potentially risky procedures and so I think that's kinda what's driving some of the limitation." [ID#107, Allergist, private practice]<br><br>"So we had to you know, take a day where we weren't giving allergy shots, where we had rooms that were available that we could use for this, dedicate staff time for this." [ID#103, Allergist, private practice]<br><br>*Dedicated staff*

	"I think dedicating a person in a, that may not be all they do in the beginning but at least having a go to person that will help answer the questions for the patients get them signed up on the REMS programme, go ahead and start that insurance approval process and get all of that done because I think it's best to have one person that is familiar with the process and all of these checks, making sure that the you know the product is in the office, that the patient's product is being delivered to the patient, that all of that's taking place, I think having one dedicated person to handle that is best." [ID#201, Nurse practitioner, private practice]<br><br>*Space*<br><br>"We have like a dedicated room that we have for our Palforzia patients as we're doing the up-dosings." [ID#106, Allergist, private practice]<br><br>"I'm in a single specialty group, what we've elected to do is have just certain dedicated offices treat our Palforzia patients, you know like they're coming to my office but you know, two or three offices are feeding it because you know, we're not able to do it in some of the offices just because of the physical plan." [ID#101, Allergist, private practice]<br><br>*Scheduling*<br><br>"We've had to designate certain days in the clinic that we can particularly provide the initial dose escalation appointment, because of some space limitations and staffing limitations. So we've kind of had to you know … dictate when this may happen based on our, clinic space and staffing." [ID#107, Allergist, private practice]<br><br>*Patient communication*<br><br>"So what's worked so far is making sure the patients understand from the beginning, what they're getting themselves into and I usually, when we train our patients and they are able to verbalise everything and knowing how long they're gonna be updosing and how they're gonna stay on the maintenance dose for a very long time, and them understanding that from the beginning makes it easier for us to prescribe and be able to get the patient on therapy. I think it just comes down to patient understanding and the family members, that you know are getting their kid in therapy, that makes it easier for us." [ID#203, Nurse practitioner, private practice]
**Training and resources**	*Shared resources*<br><br>"I think the other thing and again I know every hospital or private practice, every allergy group's gonna have their own paperwork but I think maybe if Aimmune had provided like sample charting and saying, "Hey, this is just what ..", you know, uh show what like a few other clinics have done and just say, "Hey, these are some of the ways that people have charted" and you know, they can take it and run with it, they can take it and use it, they can take it and modify it, you know." [ID#202, Nurse practitioner, academic institute]<br><br>*Tailored resources*

	"…with the small practices where we're not having the volume of patients, we don't have the ancillary staff to be dedicated to just do this, I think maybe we could use more guidance in them telling like small practices versus big practices of what they think the best approach would be." [ID#104, Allergist, private practice]<br><br>*Patient materials*<br><br>"I guess one of the things that I think would be helpful in those booklets is giving like a better sense of the schedule it's kind of more abstract, the way it's described in the the booklets and I think that would be helpful like for patients to kind of get a better understanding of the up-dosing schedule." [ID#106, Allergist, private practice]<br><br>*Staff and practice materials*<br><br>"If you know, more of the like prepared sheets of information of the dose escalation of time given, observations, you know, a form that we would just kind of check off more would be probably nicer, nice to have at least." [ID#104, Allergist, private practice]<br><br>"I think all of that, other practices would appreciate I think, 'cause it was a lot, it was a lot to get going, a lot of stuff that you know, Aimmune doesn't provide all the questions and frequently asked questions forms to give patients and consent forms and things that we had to develop and I think we're doing a good job." [ID#103, Allergist, private practice]
**Reflections**	*Positive thoughts about Palforzia*<br><br>"Overall, I would say it's been a positive experience, we're very excited that we can finally offer something that could help to mitigate some of the risk that is involved with peanut allergy and improve quality of life for patients and their families." [ID#107, Allergist, private practice]<br><br>*HCP reported patient reflections on Palforzia*<br><br>"Families have been very appreciate that they're able to be on peanut and that their anxiety levels go down because they're not worried about their, their child having a reaction, they can go out to restaurants now and, and eat at, at a restaurant that might have otherwise been off limits to them for some reason." [ID#105, Allergist, academic institute]<br><br>*Recognition of trade-off between implementation challenges and benefits of treatment*<br><br>"I would definitely say there's positives, I would say that there's more negatives than positives, but I would never stop doing this treatment, I mean I wholeheartedly believe OIT changes lives so I'm all for doing it, I just think the process could be better." [ID#102, Allergist, private practice]
