# Supplementary material for: Qualitative interviews to understand health care providers’ experiences of prescribing licensed peanut oral immunotherapy
Source: BMC Res Notes. 2022 Aug 8;15:273. doi: 10.1186/s13104-022-06161-6 (PMC9358114; doi:10.1186/s13104-022-06161-6)
Supplement: Supplementary file 5 — Additional file 5: Table S4. Delivering Palforzia during the COVID-19 pandemic (Theme 4). Table presenting additional quotes to support the data presented in the manuscript (Theme 4). [file 13104_2022_6161_MOESM5_ESM.pdf]

Additional file 5

Supplemental Table 4. Delivering Palforzia during the COVID-19 pandemic (Theme 4)

Sub-theme	Selected quotes
**Challenges of delivering Palforzia COVID-19 pandemic**	*Delays to implementation*<br><br>"Yes, you know we were hoping to start in the spring, soon after the product had been approved and available for distribution but due to the pandemic, you know, we pushed that back and did not start until the summer." [ID#107, Allergist, private practice]<br><br>*Clinic closures*<br><br>"Our clinic was closed for two months during COVID so we were closed in April and May, so we started again seeing patients again in June. So in June, after that point, you know, we make sure everything was in place and that we are not going to close again." [ID#108, Allergist, academic institute]<br><br>*Reduced staffing*<br>"And then of course when COVID hit, we had front desk and nurses that were furloughed so now, we're you know on, in some offices, a skeleton crew and you're talking about now offering this product that really is gonna need additional attention and additional staffing and we just didn't have it." [ID#201, Nurse practitioner, private practice]<br><br>*Staff reluctance*<br>"So I would say that they've definitely been [sighs] slow adopters when we started OIT… so we have not gotten everybody on board yet although I would say that probably some of the reasons why we haven't got everyone on board probably is more related to COVID at this point in time, than that people don't believe in the process." [ID#202, Nurse practitioner, academic institute]<br><br>*Preventative measures*<br>"Our clinic has done very well in terms of being able to socially distance within the clinic, cleaning procedure, sanitisation procedures, screening of patients before they're coming in and so that's kind of created what we call a COVID safe environment." [ID#107, Allergist, private practice]<br><br>*Patient and family interest in OIT during the pandemic*<br><br>"Anything that motivated the patient to be on Palforzia has been superseded by the potential risk of the contagion. And the people that are at risk from peanut allergy, the respiratory people, those are also at risk for COVID." [ID#102, Allergist, private practice]<br><br>"I mean the families that have been interested in doing it, I think, I think we've only had one family that said they don't want to start until COVID is over and other than that, the ones that have wanted to do it, have not found COVID to be uh, a deterrent." [ID#202, Nurse practitioner, academic institute]<br><br>*Exacerbation of clinic factors*

	“Yeah, we’ve really had to even have you know, a more dedicated space for the patient because a lot of patients don’t want to sit in a waiting room. The plan originally was we would give them a dose, have them go sit in the waiting room, come back for their next dose and they’d be in and out of the waiting room, people don't want to be sitting in a waiting room right now so it took you know, even more dedicated space that had to be for that patient, that you couldn’t see anyone else.” [ID#103, Allergist, private practice]<br><br>“Initially I would have two patients that were coming in on the same day, actually I had three patients who were coming in on the same day, so we had to switch it out 'cause we wanted to have specific rooms because they’d be here for an hour so … we kinda moved them where we have one, I’m not seeing those patients at the same time anymore.” [ID#203, Nurse practitioner, private practice]
**Unexpected opportunities**	*Time to learn and scale up slowly*<br>“It’s very labor intensive and time intensive and it’s the kind of thing we have to prepare for so actually, if anything, the pandemic did give us more time to get prepared and set-up and have our, our procedures in place.” [ID#103, Allergist, private practice]
